# Mitochondrial Dysfunction as a Novel Target for Neuroprotective Nutraceuticals in Ocular Diseases

**DOI:** 10.3390/nu12071950

**Published:** 2020-06-30

**Authors:** Chun-Ping Huang, Yi-Wen Lin, Yu-Chuen Huang, Fuu-Jen Tsai

**Affiliations:** 1 School of Chinese Medicine, College of Chinese Medicine, China Medical University, Taichung 404, Taiwan; hcpnttw@gmail.com; 2 Graduate Institute of Acupuncture Science, College of Chinese Medicine, China Medical University, Taichung 404, Taiwan; yiwenlin@mail.cmu.edu.tw; 3 Department of Medical Research, China Medical University Hospital, Taichung 404, Taiwan; 4 Children’s Hospital of China Medical University, Taichung 404, Taiwan; 5 Department of Medical Genetics, China Medical University Hospital, Taichung 404, Taiwan

**Keywords:** nutraceuticals, neuroprotection, ocular diseases, mitochondrial dysfunction

## Abstract

The eyes require a rich oxygen and nutrient supply; hence, the high-energy demand of the visual system makes it sensitive to oxidative stress. Excessive free radicals result in mitochondrial dysfunction and lead to retinal neurodegeneration, as an early stage of retinal metabolic disorders. Retinal cells are vulnerable because of their coordinated interaction and intricate neural networks. Nutraceuticals are believed to target multiple pathways and have shown neuroprotective benefits by scavenging free radicals and promoting mitochondrial gene expression. Furthermore, encouraging results demonstrate that nutraceuticals improve the organization of retinal cells and visual functions. This review discusses the mitochondrial impairments of retinal cells and the mechanisms underlying the neuroprotective effects of nutraceuticals. However, some unsolved problems still exist between laboratory study and clinical therapy. Poor bioavailability and bioaccessibility strongly limit their development. A new delivery system and improved formulation may offer promise for health care applications.

## 1. Introduction

Retinal neurodegeneration is one of the major causes of visual impairment and is highly associated with atrophy or cell death of the retina in ocular diseases, such as glaucoma, age-related macular degeneration (AMD), and diabetic retinopathy (DR) [1]. The metabolic rate of the visual system is higher than that of others within the brain, and further disruptions in the metabolic homeostasis can lead to vulnerability of the retina [2] In this review, we endeavored to focus on the neuropathies of retinal cell types induced by metabolic impairment, as well as the potential neuroprotective nutraceuticals based on evidence from animal experiments and clinical studies.

### 1.1. The High-Energy Demands of the Retina

The process of visualizing information is achieved by transmitting light into electrical impulses, triggered mainly by rods and cones, through the optic nerve to the brain. Bipolar cells and amacrine cells collect the signals from photoreceptor cells and synapse with retinal ganglion cells (RGCs). RGCs are a type of neuron and propagate action potentials (AP) to axons. The axons form the optic nerve and project to the lateral geniculate nucleus (LGN), the medial geniculate body, and superior colliculus. There are five neuronal cell types: photoreceptor, horizontal, bipolar, amacrine, and ganglion cells in the retina [3]. In the visual system, phototransduction, neurotransmitter utilization, protein synthesis transport, and repolarization-after-depolarization are energy-dependent [2]. The amount of oxygen and nutrient consumption in the retina is vast because of the intricate neural networks. Mitochondria are the major endogenous sources of adenosine triphosphate (ATP) and reactive oxygen species (ROS) in mammalian cells. Mitochondria are dynamic organelles and mitochondrial integrity is maintained by fusion, fission, mitophagy, and biogenesis. Fusion stimulates the formation of elongated mitochondria to produce more ATP. Excessive accumulation of ROS causes fission and triggers mitochondrial fragmentation. Damaged mitochondria are further self-destructed for turnover by the mitophagy pathway and then biogenesis replenishes number and/or mass of de novo mitochondria [4,5,6]. Energy metabolism overexertion causes an imbalance between the generation and elimination of ROS. Furthermore, excess ROS causes oxidative stress, which leads to retinal neurodegeneration, visual loss, and eventual blindness [7].

### 1.2. Disruptions of the Metabolic Homeostasis Cause Mitochondrial Dysfunction in the Retina

Under normal physiological conditions, mitochondrial ROS (mtROS) is quickly scavenged by manganese superoxide dismutase (SOD2), maintaining metabolic homeostasis. Overproduction of mtROS damages the cells by increasing levels of oxygen free radicals and further causes lipid peroxidation, protein carbonylation, and DNA breakage [8], especially mitochondrial DNA (mtDNA) without protection by histones and repair mechanisms [9]. A burst of ROS is a primary event in glutamate-induced neurotoxicity [10], and vice versa in *optic atrophy gene 1* (*OPA1*) mutation animal models [11]. Furthermore, activation of ionotropic glutamate receptors raises Ca^2+^ entry into both the cytosol and mitochondria, and induces the process of neuronal death by apoptosis or necrosis [12,13]. The increasing number of free radicals also attack lipoproteins and form intracellular lipid deposits to alter autophagy or phagocytosis in the retina [14]. The term ‘mitochondrial optic neuropathy’ (MON) suggested the need to investigate the pathogenetic role of the mitochondria. There are three major acquired etiologies, including toxic, nutritional, and metabolic insults which cause MON [15]. Moreover, chronic and metabolic disorders, such as dyslipidemia or hyperglycemia, disturb the homeostasis of the mitochondrial dynamic and develop neurodegeneration as an early event in the pathogenesis of glaucoma [16], AMD [14] and DR [6,17].

### 1.3. The Characteristics of Mitochondrial Dysfunction in Retinal Neurodegeneration

Glucose and lipid metabolism are mostly dependent on the mitochondria, therefore, mitochondrial abnormality is seen in metabolic syndromes [18]. Mitochondrial oxidative stress alters the delicate balance between fusion and fission, mitochondrial fragmentation, and further increases the number of autophagosomes and accumulation of dysfunctional mitochondria [19,20,21]. In parallel, it accompanies the cytosolic increase in the activity of the polyol pathway, advanced glycation end products (AGEs), and protein kinase C (PKC) activation [22,23]. The susceptibility to oxidative stress is closely associated with the distribution and amount of mitochondria in RGCs [17,24]. The anatomical structure of RGCs is another important factor which causes optic neuropathy. The anterior part of the axon is unmyelinated and has slower conduction velocities. That means more energy consumption is necessary to maintain the generation and propagation of AP. These neuropathic phenotypes of RGCs include loss of dendritic arborization, axonal loss, optic nerve atrophy, and retinal nerve fiber layer thinning to cause clinical signs of reduction in visual evoked potential (VEP) and optic nerve degeneration [17].

In the retina, mitochondria are also found to be abundant at the distal ends of the retinal pigment epithelium (RPE) and photoreceptors, especially the basement membrane, cilium, and outer feet [25]. Mitochondrial dysfunction of a coordinated ecosystem between RPE and photoreceptors has recently been described. Photoreceptors utilize glucose by glycolysis and convert it to lactate, which transports back to the RPE cells as a fuel through oxidative phosphorylation [26]. Conditional knockout of *Sod2* in the RPE elevates levels of oxidative stress and dysfunctional mitochondria in both RPE and photoreceptors [27]. In addition, the metabolism of photoreceptors becomes maximal to generate dark current at night [28]. Therefore, photoreceptors are the major site of ROS generation and show apoptosis in the early stages of diabetes [29] and atrophy in the later stages of AMD [17]. In brief, the high energy requirement and electrophysiological function of retinal cells make them more vulnerable to oxidative stress.

### 1.4. Nutraceuticals as Neuroprotectants for Retinal Neurodegeneration

Metabolic impairments cause numerous retinal manifestations in chronic progression. This results in irreversible damage of retinal cells if oxidative stress is not reduced with treatment. Some clinical medications or surgeries are approved to treat ocular diseases. However, these therapies are used for the late stages of disease progression. There is a clear need for new strategies to act at the molecular or cellular target to prevent the development of the disorder. Mitochondrial dysfunction has been shown to be one of the early events in retinal neurodegeneration [30,31]. Targeting the mitochondrial function brings the promise of new options. Nutraceuticals are believed to target multiple pathways and attenuate the progression of neuronal destruction through mitochondrial dysfunction [32,33]. Therefore, nutraceuticals could be considered as positive neuroprotectants of retinal cells. Here, we summarize the retinopathy progression related to mitochondrial dysfunction and the effects of nutraceuticals with improved retinal neurodegeneration as below.

## 2. Mitochondrial Dysfunction in the Pathogenesis of Ocular Diseases

### 2.1. Glaucoma

Glaucoma is the second leading cause of blindness worldwide and is characterized by the progressive degeneration or loss of RGCs [34]. Most glaucoma cases are classified into primary open-angle glaucoma (POAG) and angle-closure glaucoma (ACG) caused by an increase in the intraocular pressure (IOP). Mitochondrial dysfunction in the trabecular meshwork may impair their cytoarchitecture and lead to alteration in the drainage of aqueous humor, further raising the IOP [16,35]. IOP–induced stress and strain are biomechanical factors of damage in the lamina cribrosa and adjacent tissues [34]. It also induces metabolic stress causing mitochondrial dysfunction in mouse RGCs [36]. In some individuals with normal range IOP, particularly in Asians, they are classified as normal-tension glaucoma (NTG) [37]. The pathogenic mechanism of NTG is not fully understood; the low pressure of cerebrospinal fluid in the optic nerve subarachnoid space may cause trans-lamina cribrosa pressure difference and compress the optic nerve [38]. Numerous biomarkers related to oxidative stress are reported to be significantly higher in glaucoma patients [39]. Therefore, recent scientific literature demonstrates that mitochondrial dysfunction and oxidative stress are both a cause and consequence and play a central role in the process of glaucoma [40].

### 2.2. Age-Related Macular Degeneration

AMD is a progressive ocular disease with loss of central vision and is a major cause of visual impairment in the developed world. It is clinically classified as early-stage (formation of drusen deposits between the Bruch’s membrane and RPE) and late-stage AMD owing to atrophy of the RPE/photoreceptors (dry) or choroidal neovascularization (wet) [41]. Complement factor H (CFH) is an important component of drusen, indicating a local complement-activation at the RPE [42]. Oxidative stress suppresses the expression of CFH [43] and promotes complement system activation [44], abolishing its protective function from the lipid peroxidation product [45]. Smoking is believed to be the strongest risk for developing AMD [46] and leads to oxidative stress and complement activation, resulting in the endoplasmic reticulum (ER) stress-mediated lipid accumulation [47]. Initial RPE mitochondrial abnormalities have been revealed in AMD patients [48]. Furthermore, mtDNA damage is found in the macular and peripheral RPE of AMD human samples [49]. Therefore, AMD could be seen as a progressive neurodegenerative disease primarily causing damage to mtDNA and further affecting the mitochondrial function of RPE [50].

### 2.3. Diabetic Retinopathy

**Figure 1:**
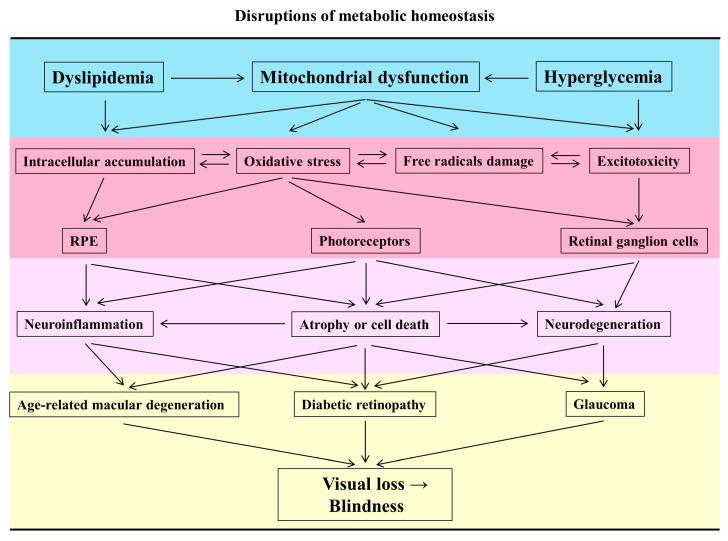
Different pathogenetic mechanisms lead to ocular diseases. Arrow indicates pleiotropic factors, as described above, and how they interact with the retinal cells that contribute to eye diseases.

DR is one of the most common complications of diabetes and remains the leading cause of vision loss among working-age adults in developed countries [51]. It is traditionally characterized as a microvascular disease [52] and has recently been recognized as a disruption of the interdependence between multiple retinal cell-types, causing neurodegeneration at the endpoint [53,54]. Hyperglycemia and poor glucose control are fundamental in the development of DR [55,56]. RGCs, amacrine cells, and photoreceptors have an increased apoptotic rate at the early stages of DR development in humans [57,58,59]. This apoptotic death causes pericentral macular thinning of both the inner retinal layers and the nerve fiber layer (NFL), otherwise, hypertrophy (swelling) of Müller cells increases the thickness of the inner nuclear layer (INL) [60,61,62]. The detailed mechanisms of apoptosis in the development of DR are still not clear [63]. There are multiple factors involved in the pathogenesis, including AGEs, free radicals, excitotoxicity, and mitochondrial damage as mentioned above. Finally, hyperglycemia-induced metabolic stress may initiate a vicious cycle to amplify mitochondrial dysfunction, and further accelerate the apoptosis of retinal cells [64,65]. Severe loss of retinal cells causes failures in orchestrating intimate communication and promotes compensatory over-angiogenesis in proliferative DR [66,67] (Figure 1).

## 3. Neuroprotective Effects of Nutraceuticals in Animal Studies and Clinical Trails

### 3.1. Resveratrol

Resveratrol is a plant polyphenol found in grapes and red wine [68], and is reported to improve mitochondrial function by activating SIRT1 and peroxisome proliferator-activated receptor-γ coactivator 1α (PGC-1α) [69]. It is also effective for age-related ocular diseases through anti-oxidant and anti-inflammatory properties [70]. Evidence demonstrates that resveratrol raises the survival of RGCs [71] and protects the loss of dendrite complexity [72] in glaucoma experimental models. Reports have indicated that resveratrol causes a reduction in IOP in steroid-induced ocular hypertension rats [73] and normal normotensive rabbits [74]. Resveratrol has neuroprotective effects through suppressing apoptosis-related molecule activator protein 1 (AP-1) [75] and elevating neuroprotective factors such as leukemia inhibitory factor (LIF), brain-derived neurotrophic factor (BDNF), oncostatin M (OSM), cardiotrophin 1 (CT-1), and cardiotrophin-like cytokine (CLC) [76] in light-induced retinal degeneration mouse models. It has also demonstrated benefits in alleviating hyperglycemia, oxidative biomarkers, vascular damage, anti-inflammation, anti-apoptosis, and reduction in the thickness of retinal layers in DR animal models. The proposed mechanisms include reduction in nuclear factor κB (NF-κB), paraoxonase 1 (PON1), and vascular endothelial growth factor (VEGF) [77,78,79,80].

Resveratrol has numerous beneficial effects on anti-cancer, cardiovascular diseases, obesity, diabetes, and neurological disorders in humans. It is reported to be safe at a dose of 1 g or more per day, however, the major obstacle for clinical therapy is the rapid metabolism and poor bioavailability [81,82,83]. Until now, research on resveratrol has been limited to animal models and in vitro experiments in ocular diseases [70,84]. Case report observations have shown resveratrol based nutritional supplements have benefits to improve RPE functions in AMD patients [85]. In addition, a recent double-blind randomized control trial indicates resveratrol notably reduces muscle fat and improves mitochondrial function in diabetes type 2 (T2D) patients [86]. Further investigations into the retino-protective effects of resveratrol should include more clinical studies. 

### 3.2. Quercetin

Quercetin is a dietary flavonoid compound found in fruits, vegetables and beverages [87]. It has a substantial antioxidant ability to scavenge ROS [88] and ameliorates mitochondrial dysfunction through an AMP-activated protein kinase (AMPK)/SIRT1 signaling pathway [89,90]. An increasing number of studies show that quercetin reduces ROS [91,92], mitochondrial membrane potential (ΔΨm) and has anti-apoptotic effects on RGCs [93]. Zhou and colleagues recently report quercetin alleviates the excitability of RGCs through increased miniature GABAergic neurotransmission and decreasing miniature glutamatergic neurotransmission [94]. On the other hand, some studies have demonstrated that quercetin has an inhibitory effect of heat shock protein 72 (HSP 72) in RGCs [95,96,97]. Quercetin has neuroprotective effects of retinal layers [98] and cytoprotective effects of photoreceptor, RPE and RGCs through inhibiting activity of AP-1 pathway [99] in light-induced retinal degeneration rodent models. It attenuates hyperglycemia [100] and dyslipidemia [101], and also has anti-retinal oxidative stress, anti-neuroinflammation and anti-apoptosis protective effects in diabetic animal models [102].

Clinical trials on quercetin have shown multiple effects, such as anti-inflammatory effects through the reduction in plasma C-reactive protein [103] or oxidative stress markers [104], anti-cancer effects, and cancer chemoprevention [105,106,107]. In a recent cohort study with a 15-year follow-up, dietary intake of quercetin was shown to reduce the prevalence of any AMD (OR: 0.76; 95% CI: 0.58, 0.99) [108]. However, the lack of clinical data limits its application in ocular diseases; thus, more clinical studies are required in the future.

### 3.3. Xanthophylls (Lutein and Zeaxanthin)

Lutein and zeaxanthin stereoisomer are oxygenated carotenoids (xanthophylls) and are present at the macula as macular pigments [109]. Xanthophylls cannot be synthesized in humans and their supplements depend on dietary sources. They are abundant in various foods such as spinach, egg yolk, and wolfberry [110]. Xanthophylls play a key role in ROS scavenging and have anti-inflammatory and neuroprotective functions [110,111,112]. They are cleaved by β,β-carotene 9′,10′-oxygenase 2 (BCO2), however, inactivity of human BCO2 causes carotenoid accumulation [113]. This phenomenon may be an important mechanism for protecting the macula from short-wavelength light-induced damage [114]. Lutein has multiple benefits via anti-apoptosis [115], antioxidant [116] and reducing ER stress [117] in the retina. Recent studies demonstrate that xanthophylls could upregulate carotenoid metabolic genes and also improve mitochondrial biogenesis in primate animal models [118,119].

Many studies show multi-ingredient formulations for individuals could increase the concentrations of lutein or xanthophylls in the plasma and macular pigment (reviewed by Bernstein et al. [120]). Epidemiologic studies support lutein for the prevention of developing AMD in the early or intermediate stage [121,122]. It is also reported that lutein/zeaxanthin may be protective against late AMD [123]. A recent systematic review reported that there are at least 47 publications from 1946 to October 2016 and its conclusions show a strong relationship between lutein/zeaxanthin supplementation and evaluation of both macular pigment density and visual function [124]. There are controversial results of lutein and zeaxanthin in an Age-Related Eye Disease Study 2 (AREDS2) [125]. Their data showed that AREDS2 formulation in primary analyses did not prevent process of advanced AMD. However, when participants were limited to those with the lowest dietary intake of lutein + zeaxanthin, results of exploratory subgroup analyses showed a protective effect for progression to advanced AMD (HR: 0.74; 95% CI, 0.59–0.94; *p* = 0.01). These inconsistent results of xanthophylls in clinical trials need further design approaches to confirm their benefits.

### 3.4. Omega-3 Fatty Acid

Docosahexaenoic acid (DHA, C22:6) and eicosapentaenoic acid (EPA, C20:5), belonging to omega-3 (α-linolenic acid, n-3) polyunsaturated fatty acids (PUFAs), are required for membrane organization and cell integrity. PUFAs intake from dietary supplementation is essential as mammals lack the enzymes for its generation [126]. Omega-3 PUFAs improve the mitochondrial dysfunction by upregulating mitochondrial biogenesis, ATP production, and dissipating the proton gradient uncoupling proteins (UCPs) gene expression in vivo [127,128,129]. DHA is enriched in the retina, where it has both structural and neuroprotective functions, and is converted by lipoxygenase (LO) to 10,17S-docosatriene (neuroprotectin D1, NPD1) under oxidative stress conditions [130]. Furthermore, NPD1 inhibits pro-inflammatory and apoptotic gene expression, and consequently promotes the survival of photoreceptors [131]. Dietary manipulation of omega-3 PUFAs lowers IOP in aged rats and is associated with a significant increase in the outflow facility and a decrease in ocular rigidity [132]. Another study showed that a dietary combination of omega-3 and omega-6 PUFAs are more effective for preventing retinal cell structure and decreasing the glial cell activation [133]. An omega-3 fatty acid diet has been shown to have a retinal protective function in the AMD-like retinal lesions [134] and type 2 diabetic mice [135]. A recent study indicated that omega-3 PUFAs reduce lipofuscin granule formation and protect the photoreceptor layer. Its mechanism may involve an increase in the myelin basic protein (MBP), myelin proteolipid protein (MPP), myelin regulatory factor-like protein (MRFLP), and glial fibrillar acidic protein (GFAP) expression [136].

Omega-3 PUFAs intake is associated with a 30% decrease in the development of central geographic atrophy (CGA) and neovascular AMD [137]. Dietary supplementation with 4 g of omega-3 PUFAs for 6 months increases the serum omega-3 in patients with dry AMD by an average of 7.6%, however, there are no statistically significant changes in the retinal function of visual acuity or ERG [138]. The modified AREDS formulation also has a similar outcome in that the addition of omega-3 PUFAs in primary analyses did not reduce risk of progression to advanced AMD [125]. A recent AREDS2 study showed similar results, where omega-3 PUFAs did not demonstrate any significant benefit in the reduction in their risk for progression to late AMD in participants with CFH or age-related maculopathy susceptibility 2 (ARMS2) risk genotype [139]. Conversely, increasing dietary PUFA, rather than saturated FA, is associated with a reduced likelihood of the presence and severity of DR [140]. A Mediterranean diet with omega-3 PUFAs (≥500 mg/day) supplements also showed a 48% relatively reduced risk in the incidence sight-threatening DR in individuals with type 2 diabetes [141].

### 3.5. Curcumin

Curcumin is a polyphenol extracted from turmeric (*Curcuma longa*), which is used as a spice and as a traditional herbal medicine in Asia. It is a hydrophobic molecule and is almost insoluble in water (approximately 30 nM). Curcumin has strong free radical scavenging activity due to its functional groups and sequentially improves mitochondrial functions through the nuclear factor erythroid 2-related factor 2 (Nrf2) [142]. However, its poor solubility and low bioavailability have limited the clinical applications of curcumin (see a recent review in detail [143]). New strategies, including liposomes and nanoparticle carriers, or modified formulations may be an ideal approach to deliver curcumin to the lesions. For example, Davis et al. developed a curcumin nanocarrier combined with D-α-tocopherol polyethene glycol 1000 succinate (TPGS), a non-ionic surfactant, and Pluronic F127, a difunctional block copolymer surfactant, which increased curcumin solubility by 400,000 times and that enhanced curcumin transport across ocular barriers. A topically administered curcumin nanocarrier has neuroprotective effects of retinal cells in vitro and in vivo [144]. In addition, Cheng and colleagues recently reported a dual-drug delivery system which consisted of thermosensitive chitosan–gelatin-based hydrogel containing curcumin-loaded nanoparticles and latanoprost, which release medicine and was extended to 7 days. Treatment with curcumin-containing hydrogel effectively decreased the oxidative stress-mediated damage in trabecular meshwork cells [145].

Clinical application of curcumin has been broadly discussed in multiple malignant diseases [146]. However, there are few studies which report that curcumin has clinical benefits for eye disorders. Improved formulation may overcome this problem. One example is that oral administration of a curcumin-phospholipid delivery system is effective in the management of central serous chorioretinopathy (CSCR). The results show administration of curcumin significantly improves visual acuity and retinal thickness [147]. A recent review examined this issue and the authors categorize three broad formulation strategies to enhance bioavailability and metabolism of curcumin [148]. These well-designed formulations require more clinical trials to confirm their substantial benefits.

### 3.6. Crocetin

Crocetin is an apocarotenoid, which is found both in the saffron crocus (*Crocus starus* L.) and in gardenia fruit (*Gardenia jasminoides* Ellis) [149,150]. Saffron and its components (crocetin, crocins, and safranal) have therapeutic properties of liver, nervous and cardiovascular systems, including anti-oxidant, anti-inflammatory, and anti-apoptotic properties [151,152]. Crocetin rescues disruption of the ΔΨm induced by tunicamycin or hydrogen peroxide (H_2_O_2_) in vitro and has protective effects against retinal degeneration in vivo [153]. It is reported to inhibit oxidative stress via mitogen-activated protein kinases (MAPK), extracellular signal-regulated protein kinases (ERK), c-Jun N-terminal kinases (JNK), p38, and the redox-sensitive NF-κB and c-Jun pathway in an ischemia/reperfusion (I/R) mouse model [154]. A hydrophilic saffron extract standardized to 3% crocin reduces higher IOP values and activated microglia cells [155]. Saffron has been shown to have beneficial effects for ocular diseases in clinical studies (see a recent review [156]). However, a recent clinical study shows short-term saffron supplementation had no significant effects on the visual acuity and focal ERG in Stargardt disease/fundus flavimaculatus (STG/FF) patients with ATP binding cassette subfamily A member 4 (ABCA4) gene mutations [157].

### 3.7. Other Potential Nutraceuticals

Some potential nutraceuticals, like traditional Chinese medicine, have effects including neural and mitochondrial protection. Ginkgo biloba extract (GBE) contains the flavone glycosides and terpenoids, and showed stabilization and protection of mitochondrial function in Alzheimer’s disease [158]. GBE has various effects of antioxidant, microcirculation and neuroprotection activities in ocular diseases [159,160]. A topical formulation of GBE reduces IOP elevation and accumulation of extracellular materials in dexamethasone-induced ocular hypertension rabbits [161]. GBE administration has been shown to improve pre-existing visual field damage [162] and visual acuity analyzed by Humphrey Visual Field (HVF) [163] in patients with NTG. However, there is a recent study that showed no effect on mean defect or contrast sensitivity in Chinese patients with NTG [164].

Danshen (*Salvia miltiorrhiza*) is used for treating hyperlipidemia, acute ischemia, and stroke in traditional Chinese medicine [165]. Danshen extracts increase the levels of glutathione (GSH) and reduce the levels of malondialdehyde (MDA) in the eye tissues of hyperglycemic rats [166]. Salvianolic acids are a natural compound extracted from Danshen and more than 10 different salvianolic acids have been identified to date. Salvianolic acid A and B are the most effective and abundant compounds [167]. Salvianolic acid A has anti-oxidative stress potential, through the activation of Nrf2 and hemeoxygenase-1 (HO-1) expressions in RPE cells [168]. It also has an anti-angiogenesis function through the downregulation of cylindromatosis (CYLD) signaling pathways in choroidal neovascularization (CNV) mice [169]. A recent study showed that salvianolic acid A improved the mitochondrial function of high glucose-injured Schwann cells and diabetic peripheral neuropathy (DPN) in KK-A^y^ diabetic mice via upregulation of nuclear Nrf2 expression [170]. Salvianolic acid B has been reported to protect against oxidative injury through Nrf2 and glutaredoxin 1 (a thiol repair enzyme, Grx1) in primary human RPE cells [171]. A multiple-formula containing Danshen, notoginseng, and borneol (Compound Danshen Dripping Pill, CDDP) significantly improved the best corrected visual acuity and retinal pathogenesis in non-proliferative diabetic retinopathy (NPDR) patients [172]. Another study showed that CDDP significantly improved fluorescence fundus angiography and funduscopic examination parameters in NPDR patients [173].

Astragali Radix (Huangqi) is one of the most frequently used herbal medicines in traditional Chinese medicine and has a wide range of biological activities [174]. The extract of *Astragalus mongholicus* has been reported that protected oxidative damage through ameliorating activities of the mitochondrial complexes I, II, malate dehydrogenase and ΔΨm [175]. *Astragalus* polysaccharides protects mitochondria by scavenging ROS, inhibiting mitochondrial permeability transition and increasing the activities of catalase (CAT), SOD, and glutathione peroxidase (GPx) [176]. Huang and colleagues further demonstrated that *Astragalus* polysaccharides restored the imbalance of mitochondrial fusion–fission processes, activation of mitophagy, and decrease in PGC-1α expression in vivo [177]. The active compounds astragaloside IV and formononetin extracted from huangqi have also been indicated to inhibit aldose reductase (AR) and hypoxia-induced neovascularization, respectively [178,179]. These data suggest that extracts from huangqi may have therapeutic benefits for DR. Major findings are summarized in the Table 1 (animal studies) and Table 2 (clinical trials).

## 4. Conclusions

In this review, we summarize the underlying mechanisms of high-energy consumption and metabolic homeostasis that play a decisive role in the retina. The loss of balance between energy production and free radicals quenching causes oxidative stress, and further leads to mitochondrial dysfunction. The structural and functional integrity of the mitochondrion is important for maintaining the organization of retinal cells. Retinal neurodegeneration is a pathogenic result of mitochondrial dysfunction and contributes to an early stage of progression in retinal metabolic disorders.

A large body of evidence demonstrates that nutraceuticals target mitochondrial function to restore the mitochondrial flexibility. Some are essential nutrients and have benefits for both forming the cellular structure and scavenging ROS. Otherwise, multiple nutraceuticals with the potential for improving mitochondrial integrity have been reported in animal studies. Unfortunately, poor bioavailability and bioaccessibility limit their current therapeutic use. A new delivery system and improved formulation may bridge the gap between laboratory study and clinical treatment [180]. Future clinical trials require an additional focus on the next generation of nutraceuticals to confirm their health benefits.

## Figures and Tables

**Table 1:** Mechanisms of ocular neuroprotection by nutraceuticals in animal models.

Diseases	Nutraceutical	Effects	Mechanisms	Animal Models	Refs.
Glaucoma	Resveratrol	Neuroprotection	Apoptosis ↓	Intracameral injection of hyaluronic acid-induced rats	[71]
	Resveratrol	Neuroprotection	BiP↑, CHOP↑, XBP-1↓	Optic nerve crush experimental mice	[72]
	Resveratrol	IOP reduction	Binding through A1R	Steroid-induced ocular hypertension rats	[73]
	Resveratrol and quercetin	IOP reduction	Synergic effects	Normal normotensive rabbits	[74]
AMD	Resveratrol	Prevent retinal degeneration	ONL↑, ERG↑, Apoptosis↓, AP-1↓, SIRT1↑	Light-induced retinal degeneration mice	[75]
	Resveratrol	Neuroprotection	ONL↑, ERG↑, LIF, BDNF, OSM, CT-1 and CLC↑	Light-induced retinal degeneration mice	[76]
DR	Resveratrol	Alleviate oxidative stress	BG↓, BW↑, SOD↑, 8-Isoprostane↓, GSSG/GSH↓, NF-κB↓, Apoptosis↓, ONL↑	STZ-nicotinamide-induced DR rats	[77]
	Resveratrol	Anti-oxidative stress	AOPP↓, MDA↓, TOS↓	STZ-induced type 1 diabetes rats	[78]
	Resveratrol	Anti-inflammation and retinal protection	BG↓, BW↑, AGEs↓, Insulin↑, Apoptosis↓, PON1↑, Ox-LDL↓, IL-1β, IL-6, TNF-α, VEGF, IFN-γ and MCP-1↓	STZ-induced diabetes rats	[79]
	Resveratrol	Retinal vascular protection	BG↓, Pericytes↑	STZ-induced diabetes mice	[80]
Glaucoma	Quercetin	Neuroprotection	ERG↑, RGC survival↑, Apoptosis↓, ΔΨm↑	Chronic ocular hypertension rats	[93]
	Quercetin	Neuroprotection	GABAergic inhibitory neurotransmission↑, glutamatergic excitatory neurotransmission↓, excitability of the RGCs↓	Electrocoagulation of the superior scleral vein rats	[94]
AMD	Quercetin	Neuroprotection	Thickness of whole retina↑, Apoptosis↓, Inflammation↓	Blue light-induced damage mice	[98]
	Quercetin	Neuroprotection	ERG↑, ONL↑, Phagosomes in RPE↑, AP-1↓	Light-induced retinal degeneration rats	[99]
DR	Quercetin	Neuroprotection	GSH↑, SOD↑, Thickness of whole retina↑, ONL↑, INL↑, TNF-α↓, IL-1β↓, Apoptosis↓, AQP4, GFAP and caspase-3↓	STZ-induced diabetes rats	[102]
	Lutein or DHA	Neuroprotection	Thickness of whole retina↑, ONL↑, INL↑, Apoptosis↓	STZ-induced diabetes rats	[112]
Retinal detachment (RD)	Lutein	Neuroprotection	ONL↑, GFAP↓, RHO↑, Apoptosis↓	Subretinal injections-induced RD rats	[115]
AMD	Lutein	Anti-oxidative stress	RPE tight junctions↑, ROS↓, SOD↑, Macrophage-related markers↓	Light-induced AMD-related mice	[116]
	Lutein and zeaxanthin	Neuroprotection	ERG↑, Apoptosis↓, p-JNK↓, Nrf2↑, GRP78, p-PERK, ATF4 and ATF6↓	Light-induced damage mice	[117]
DR	Wolfberry	Retinoprotection	Levels of zeaxanthin and lutein↑, SRB1↑, GSTP1, BCO2, and AMPK-α2↑, HIF-1α, VEGF, and HSP↓, Mitochondrial copy number↑, Citrate synthase activity↑, PGC-1α, Nrf1, and TFAM↑	Leptin receptor-deficient (db/db) type 2 diabetic mice	[118]
Glaucoma	Omega-3 PUFAs	IOP reduction	IOP↓, Aqueous outflow↑	Age-induced IOP increase rats	[132]
	Omega-3 and omega-6 PUFAs	Anti-inflammation	GFAP↓, Thickness of whole retina↑	Photocoagulation-induced IOP increase rats	[133]
AMD	Omega-3 PUFAs	Anti-inflammation	PGE2, LTB4, TNF-α and IL-6↓, PGD2↑	AMD-like retinal lesions mice	[134]
DR	Omega-3 PUFAs	Retinoprotection	ERG↑, BG↓	Leptin receptor-deficient (db/db) type 2 diabetic mice	[135]
AMD	Omega-3 PUFAs	Retinoprotection	Lipofuscin↓, ONL↑, MBP, MPP, MRFLP and GFAP↑	Aged (24-month-old) wild-type mice	[136]
Glaucoma	Curcumin	Neuroprotection	IOP↓, RGC density↑	Ocular hypertension and partial optic nerve transection rats	[144]
Retinal degeneration	Crocetin	Neuroprotection	ERG↑, ONL↑, Apoptosis↓	Light-induced damage mice	[153]
Retinal ischemia	Crocetin	Neuroprotection	GCL, INL, ONL↑, ERG↑, p-p38, p-JNK, p- ERK 1/2, p-c-Jun, p-NF-κB↓	I/R-induced retinal damage mice	[154]
Glaucoma	Saffron	Neuroprotection	IOP↓, RGC↑, Iba-1 (+) microglia↓	Laser-induced ocular hypertension mice	[155]
	GBE	Neuroprotection	IOP↓, RGC↑,	Ocular hypertension by cautery of three episcleral vessels rats	[159]
	GBE	Retinoprotection	IOP↓, Apoptotic TM cells↓	Dexamethasone–induced ocular hypertension rabbits	[161]
Diabetes	Danshen	Anti-oxidative stress	GSH↑, MDA↓	STZ-induced diabetes rats	[166]
CNV	Salvianolic acid A	Anti-angiogenesis	OX-LDL↓, Fluorescein angiography↓, VEGF↓, PDGF↓, Angiostatin↑, CYLD↓	Laser photocoagulations plus OX-LDL injection-induced CNV mice	[169]
DPN	Salvianolic acid A	Anti-oxidative stress	BG↓, Fructosamine↓, Myelin sheath thickness↑, Nrf2↑	KK-A^y^ diabetic mice	[170]
DR	Astragaloside IV	Neuroprotection	ERG↑, Apoptosis of RGCs↓, AR↓, p-ERK1/2, NF-kB↓	Leptin receptor-deficient (db/db) type 2 diabetic mice	[178]

Increase (↑), Decrease (↓), Binding immunoglobulin protein (BiP), C/EBP homologous protein (CHOP), X-box binding protein-1 (XBP-1), Adenosine receptor 1 (A1R), Optic nerve layer (ONL), Streptozotocin (STZ), Blood glucose (BG), Body weight (BW), Glycosylated hemoglobin (HbA1c), Oxidized glutathione (GSSG), Advanced oxidation protein products (AOPP), Malondialdehyde (MDA), Total Oxidant Status (TOS), Low-density lipoprotein (LDL), Interleukin (IL), Tumor necrosis factor (TNF), Interferon (IFN), Monocyte chemotactic protein (MCP), Inner nuclear layer (INL), Aquaporin-4 (AQP4), rhodopsin (RHO), phosphorylated c-Jun N-terminal kinase (p-JNK), Glucose-regulated protein (GRP78), phosphorylated protein kinase RNA-like endoplasmic reticulum kinase (p-PERK), activating transcription factor 4 (ATF4), activating transcription factor (ATF6), Scavenger receptor class B type 1 (SRB1), Glutathione S-transferase pi gene (GSTP1), Hypoxia-inducible factor-1-α (HIF-1α), Nuclear respiratory factor 1 (NRF1), Transcription factor A, mitochondrial (TFAM), Prostaglandin (PG), Leukotriene B4 (LTB4), Oxidized low-density lipoprotein (OX-LDL), Platelet-derived growth factor (PDGF).

**Table 2:** Main significant outcomes of nutraceuticals in clinical trials.

Diseases	Nutraceutical	Study Population	Study Design/Follow-Up	Results/Findings	Refs.
AMD	Resveratrol	3 cases	Case report	Restoration of structure and visual function	[85]
T2D	Resveratrol	17 subjects	Double-blind randomized cross-over study	Intrahepatic lipid↓, Intramyocellular lipid↑, Mitochondrial function (ex vivo) ↑, Metformin dose↓	[86]
AMD	Quercetin	2856 adults and 2037 followed	Population-based cohort study/15-y	Quercetin was associated with reduced odds of any AMD (OR: 0.76; 95% CI: 0.58–0.99)	[108]
AMD	Lutein/zeaxanthin	93,676 women	Cohort study/7-y	Lutein/zeaxanthin may protect against intermediate AMD (OR: 0.57; 95% CI: 0.34–0.95)	[121]
AMD	Analysis of Lutein/zeaxanthin	380 adults	Cohort study	Risk of AMD was associated with plasma concentrations of lutein/zeaxanthin (OR: 1.9; 95% CI: 0.9–3.5)	[122]
AMD	Lutein/zeaxanthin	6 publications	Meta-analysis	Dietary intake of lutein/zeaxanthin was significantly related with a reduction in risk of late AMD (RR: 0.74; 95% CI: 0.57–0.97)	[123]
AMD	Lutein/zeaxanthin, Omega-3, PUFAs, Zinc	4203 participants	multicenter, randomized, double-masked, placebo-controlled phase 3 study with a 2 × 2 factorial design/12-y	Participants were limited to those with the lowest dietary intake of lutein + zeaxanthin, results of exploratory subgroup analyses showed a protective effect for progression to advanced AMD (HR: 0.74; 95% CI, 0.59–0.94; *p* = 0.01)	[125]
AMD	Omega-3 PUFAs	1837 participants	Nested cohort study/12-y	Omega-3 PUFAs intake reduces 30% incidence to develop CGA (OR: 0.65; 95% CI: 0.45–0.92; *p* < 0.02) and neovascular AMD (OR: 0.68; 95% CI:0.49–0.94; *p* < 0.02)	[137]
AMD	Omega-3 PUFAs	17 patients	Prospective, noncomparative, descriptive pilot study	No statistically significant improvement in visual acuity and ERG; Serum omega-3 index increased by a mean of 7.6% (*p* < 0.001)	[138]
AMD	Lutein/zeaxanthin, Omega-3 PUFAs, Zinc, β-carotene	1684 participants	AREDS2/5-y	No significant interaction between supplements and genotype with improvement of progression to late AMD	[139]
DR	PUFAs	379 patients	Cohort study	PUFAs was associated with a reduction in DR severity (OR: 0.18; 95% CI: 0.06–0.59)	[140]
DR	Omega-3 PUFAs	3482 participants	Prospective, randomized clinical trial	Participants meeting the LCω3PUFA recommendation at baseline (≥500 mg/d) compared with those not fulfilling this recommendation (<500 mg/d) showed a 48% relatively reduced risk of incident sight-threatening DR, with a HR of 0.52 (95% CI, 0.31–0.88; *p* = 0.001). This association was slightly stronger for yearly updated LCω3PUFA intake (relative risk, 0.48; 95% CI, 0.28–0.82; *p* = 0.007)	[141]
CSCR	Curcuminoids and lecithin formulation	12 patients	Follow-up study/1-y	Visual acuity improvement (*p* = 0.0005 by Wilcoxon signed rank test), Reduction in neuroretinal or RPE detachment (*p* = 0.0004 by Wilcoxon signed rank test)	[147]
STG/FF	Saffron	31 patients	Randomized, double-blind, placebo-controlled study/3-y	Saffron had no detrimental effects on the visual acuity and focal ERG	[157]
NTG	GBE	27 patients	Prospective, randomized, placebo-controlled, double-masked cross-over trial	Visual fields improvement in mean deviation (*t* = 8.86, *p* = 0.0001, chi-square test) and corrected pattern standard deviation (*t* = 9.89, *p* = 0.0001, chi-square test)	[162]
NTG	GBE	332 patients	Retrospective study/1-y or more	HVF mean deviation improvement from −5.25 ± 6.13 to −4.31 ± 5.60 (*p* = 0.002)	[163]
NTG	GBE	35 patients	Prospective, randomized, placebo-controlled crossover study	No effect on mean defect or contrast sensitivity	[164]
NPDR	CDDP	57 patients	Randomized, double-dummy, double-blind study	Improvement of the best corrected visual acuity (*p* < 0.05).	[172]
NPDR	CDDP	223 patients	Randomized, double-blind, placebo-controlled clinical trial	Improvement of fluorescence fundus angiography and funduscopic examination (*p* < 0.001)	[173]

Odds ratios (OR), Confidence interval (CI), Relative risk (RR), Hazard ratio (HR).
